# Biogas Production from Sugarcane Waste: Assessment on Kinetic Challenges for Process Designing

**DOI:** 10.3390/ijms160920685

**Published:** 2015-08-31

**Authors:** Leandro Janke, Athaydes Leite, Marcell Nikolausz, Thomas Schmidt, Jan Liebetrau, Michael Nelles, Walter Stinner

**Affiliations:** 1 Department of Biochemical Conversion, Deutsches Biomasseforschungszentrum Gemeinnützige GmbH, Torgauer Straße 116, 04347 Leipzig, Germany; E-Mails: thomas.schmidt@dbfz.de (T.S.); jan.liebetrau@dbfz.de (J.L.); michael.nelles@dbfz.de (M.N.); walter.stinner@dbfz.de (W.S.); 2 Faculty of Agricultural and Environmental Sciences, Chair of Waste Management, University of Rostock, Justus-von-Liebig-Weg 6, 18059 Rostock, Germany; 3 Department of Environmental Microbiology, UFZ-Helmholtz Centre for Environmental Research, Permoserstraße 15, 04318 Leipzig, Germany; E-Mails: athaydes.leite@ufz.de (A.L.); marcell.nikolausz@ufz.de (M.N.)

**Keywords:** sugarcane waste, nutritional requirements, methane potential, degradation rates, process designing

## Abstract

Biogas production from sugarcane waste has large potential for energy generation, however, to enable the optimization of the anaerobic digestion (AD) process each substrate characteristic should be carefully evaluated. In this study, the kinetic challenges for biogas production from different types of sugarcane waste were assessed. Samples of vinasse, filter cake, bagasse, and straw were analyzed in terms of total and volatile solids, chemical oxygen demand, macronutrients, trace elements, and nutritional value. Biochemical methane potential assays were performed to evaluate the energy potential of the substrates according to different types of sugarcane plants. Methane yields varied considerably (5–181 Nm^3^·ton_FM_^−1^), mainly due to the different substrate characteristics and sugar and/or ethanol production processes. Therefore, for the optimization of AD on a large-scale, continuous stirred-tank reactor with long hydraulic retention times (>35 days) should be used for biogas production from bagasse and straw, coupled with pre-treatment process to enhance the degradation of the fibrous carbohydrates. Biomass immobilization systems are recommended in case vinasse is used as substrate, due to its low solid content, while filter cake could complement the biogas production from vinasse during the sugarcane offseason, providing a higher utilization of the biogas system during the entire year.

## 1. Introduction

Although the combined production of sugar and ethanol based on sugarcane is recognized as one of the most efficient systems for biofuels production (yield per hectare) [1], the Brazilian sugarcane industry is responsible for the generation of different types of organic waste which, in most cases, are still not being properly treated, especially from the energy point of view [2]. Based on the amount of sugarcane processed during the 2013–2014 season (653 × 10^6^ tons of cane) [3], the generation of 91 × 10^6^ tons of straw (dry basis), 169 × 10^6^ tons of bagasse (wet basis), 22 × 10^6^ tons of filter cake (wet basis), and 286–678 × 10^6^ m^3^ of vinasse are estimated.

While some of these organic wastes are directly applied as organic fertilizers on the sugarcane fields for nutrient recycling without previous energetic utilization (*i.e.*, vinasse and filter cake), the other part of the residues are mostly used as fuel in low-efficiency cogeneration systems (*i.e.*, bagasse) or even left to decay on the fields (*i.e.*, straw) due to a lack of incentives to produce bioenergy from them [4,5,6]. Non-controlled digestion of such waste on the fields may lead to the release of large amounts of methane, which may hinder the positive effect of bioenergy utilization on the climate change mitigation.

The State of São Paulo, a major sugarcane producer in Brazil, is responsible for more than 56% of the sugarcane processed in the 2013–2014 season. The state is committed to an ambitious plan to further increase the share of renewable energy from 55% to 69% until 2020, as well as to reduce the CO_2_ emissions by 20% in comparison to 2005 levels. To achieve these targets, among other measures, a biogas program was launched in 2012 to stimulate and increase the sustainable use of biomass for biogas production, including a future mandatory share of biomethane into the natural gas grid [7,8,9].

The anaerobic digestion (AD) of sugarcane waste can be considered a promising strategy, since the digestate could still be used to partially replace the mineral fertilizers on the sugarcane fields and the produced biogas could be upgraded to biomethane and sold as a new energy product by the sugarcane plants [10,11,12]. However, before being implemented on a large-scale, the AD process should be carefully evaluated, especially regarding the substrates’ characteristics, as organic matter and nutritional value, macronutrients, trace elements, and specific biogas production. Those parameters directly influence some other important process parameters, such as the pH, accumulation of inhibitors, potential macronutrients, and trace elements deficiencies, as well degradation rates.

Several authors [10,13,14] suggested that for a proper AD process a balance among the main nutrients: carbon, nitrogen, phosphorous and sulfur is necessary. If a certain substrate has a too high C:N ratio, or in another words, has nitrogen deficiency, it may negatively influence the microbial community functioning. That means a direct influence on the ability to produce enzymes that are needed to the carbon utilization, causing an incomplete conversion of the carbon contained in the substrates, resulting in lower methane yields. On the other hand, substrates that contain high levels of nitrogen can cause inhibition to the AD process via accumulation of toxic ammonia (NH_3_) produced from protein degradation or by urea conversion [15].

According to Britz *et al.* [16] and Scherer *et al.* [17], phosphorus and sulfur are also considered essential nutrients for the AD process. While sulfur is an important constituent of amino acids, phosphorus is needed for microbial growth during the formation of energy carriers ATP (adenosine triphosphate) and NADP (nicotinamine adenine dinocleotide phosphate), an important constituent of nucleic acids, as well as it plays an important role in the maintenance of an optimum pH. However, when sulfur is found in high concentrations, sulfates are reduced to sulfide by the so-called sulfate-reducing bacteria, leading to two possible inhibitions. On the one hand, due to thermodynamic advantages sulfate-reducing bacteria outcompetes methanogens for hydrogen and acetate [18,19]. On the other hand, hydrogen sulfide (H_2_S), an end product of sulfate reduction, has a toxic effect on various groups of bacteria [20]. In addition to that, when found in high concentrations, H_2_S can cause corrosion to some biogas plant parts, such as the combined heat and power units (CHP), biogas upgrading systems, and metal pipes and tanks, leading to high costs of maintenance [21].

The importance of trace elements during AD is relatively well known, especially for important enzymes and cofactors involved in different steps of the process, where special metal ions are required. Several authors [22,23] reported higher gas yields, improvements on process stability and reaching higher organic loading rates (OLR) through the supplementation of cobalt, copper, iron, molybdenum, nickel, selenium, tungsten, and zinc. While animal waste (e.g., bovine and swine manure) are expected to require less amendments, AD of energy crops, plant residues, and agro-industrial waste faces more trace element deficiencies. Therefore, it is expected that the substrate composition plays a major role for the trace elements requirements during the AD process, being an important parameter to be considered during the development of stable process with novel substrates. Additional aspects also need to be taken into account, especially regarding the trace elements’ availability for microbial uptake, mainly driven by metal speciation, pH and process temperature, applied organic loading rate, as well as the chemical processes of precipitation and complexation [23,24,25].

Furthermore, another aspect that should be taken into account when a biogas concept based on agroindustry waste is under development, is the seasonality of the crops and the feasibility of minimizing the negative effects by conserving and storing part of the substrates to be used during the offseason period. Therefore, allowing a higher utilization of the biogas system during the entire year.

To provide guidance during the designing of an AD system based on sugarcane waste, the present research performed an extensive evaluation of vinasse, filter cake, bagasse, and straw generated by plants with different production systems in the Center-South Region of Brazil, including autonomous plants, where ethanol is produced exclusively from sugarcane juice, and annexed plants, where ethanol is produced by a mixture of sugarcane juice and/or molasses (*i.e.*, waste from sugar production).

Through the analysis of several parameters, as total solids (TS), volatile solids (VS), macronutrients, trace elements, nutritional value, and biochemical methane potential (BMP). It was possible to assess the main kinetic challenges of the sugarcane waste, in terms of nutritional imbalances, energy potential, degradation rates, and proper hydraulic retention time (HRT) for the substrates’ conversion in a continuous stirred-tank reactor (CSTR) system. Moreover, in order to demonstrate how the sugarcane seasonality could be overcome, the energy synergies among the substrates were demonstrated based on the two different types of sugarcane plants.

## 2. Results and Discussion

### 2.1. Physical-Chemical Composition

#### 2.1.1. Basic Characteristics

The basic characteristics of the sugarcane waste types are presented in Table 1. Considerable differences among vinasse samples derived from sugar and/or ethanol production were found. Samples from the autonomous plant presented lower values of organic matter content in terms of COD, TS, and VS (22.1 g·L^−1^, 1.15% and 76.0% of TS), while samples from the annexed plants presented higher values for the same parameters (32.4 g·L^−1^, 3.44% and 70.6% of TS). That fact is explained, among other reasons, by the use of different feedstocks (molasses and/or sugarcane juice) during the ethanol production in these two different types of sugarcane plants. Similar values were also found previously [4] in an extensive survey about water uses in the sugarcane industry.

Filter cake presented intermediate values for TS and VS (28.9% and 74.2% of TS) in comparison to vinasse, bagasse, and straw, having an appearance similar to sludge, once it is derived from a physical-chemical treatment process that removes soluble and insoluble impurities from the sugarcane juice. Similar values were also reported during co-digestion experiments for bio-hydrogen production [26].

**Table 1:** Main characteristics of the sugarcane waste.

Parameters	Units	Vinasse				Filter Cake (*n* = 9)		Bagasse (*n* = 9)		Straw (*n* = 12)		Recommendation	
		Autonomous (*n* = 6)		Annexed (*n* = 15)									
		AV	SD	AV	SD	AV	SD	AV	SD	AV	SD	[13]	[14]
TS ^a^	% FM ^d^	1.15	±0.12	3.44	±1.11	28.9	±3.77	55.4	±4.19	76.7	±21.6	-	-
VS ^b^	% TS	76.0	±6.99	70.6	±3.84	74.2	±10.8	96.0	±2.70	86.3	±11.9	-	-
COD ^c^	g·L^−1^	22.1	±0.46	32.4	±10.0	-	-	-	-	-	-	-	-
C	% TS	37.0	±4.24	39.0	±8.61	42.7	±6.95	47.6	±2.69	43.4	±4.78	C:N 20–40:1	-
N	% TS	2.94	±0.35	2.31	±0.35	1.76	±0.24	0.41	±0.04	0.52	±0.21		-
P	% TS	0.16	±0.05	0.35	±0.12	0.60	±0.25	0.04	±0.02	0.06	±0.03	C:N:P:S 600:15:5:3	-
S	% TS	0.87	±0.49	2.12	±0.27	0.18	±0.02	0.05	±0.03	0.21	±0.06		-
Ca	mg·L^−1^	77.4	±24.3	655	±211	4139	±1667	704	±215	2981	±1656	-	100–200
Na	mg·L^−1^	16.4	±9.39	24.5	±9.58	7.75	±5.98	11.3	±8.83	37.1	±25.1	-	100–200
K	mg·L^−1^	1306	±708	6021	±565	740	±280	1651	±1036	5002	±2344	-	200–400
Mg	mg·L^−1^	173.6	±72.4	771	±177	971	±259	409	±173	1140	±404	-	75–150

^a^ total solids; ^b^ volatile solids; ^c^ chemical oxygen demand; ^d^ fresh matter; *n* = number of repetitions (each sample was analyzed in triplicate); AV = average values; SD = standard deviation.

Bagasse and straw presented relatively similar average TS and VS values (55.4% and 96.0% of TS for bagasse; 76.7% and 86.3% of TS for straw), due the fact of being mainly formed by sugarcane fibers. The only exception was one sample of straw that presented different values of TS and VS (92.4% and 68.6% of TS), possibly due to the inorganic soil particles attached to its fibers during harvesting.

#### 2.1.2. Macronutrients

The macro element contents of the sugarcane waste types are also presented in Table 1. The carbon to nitrogen ratio (C:N) of vinasse samples derived from the autonomous plant presented slightly lower average value (12:1) in comparison to the samples from the annexed plants (16:1). For both cases the values found are lower than the optimal value recommended for AD (20–40:1) [13], and could lead, in extreme cases, to process inhibition if the surplus nitrogen is converted into ammonia.

Samples of filter cake presented an average C:N ratio considered proper for AD (24:1), meanwhile bagasse and straw presented different C:N ratio profiles of 116:1 and 83:1, respectively. From the carbon and nitrogen content, a lack of nitrogen in bagasse and straw is clearly noted. However, it is noteworthy that not all of the carbon content in these lignocellulosic substrates will be bioavailable because of the high content of non-degradable lignin.

When the other macronutrients are also considered, the high sulfur content in both types of vinasse was around four, up to 10, times higher than what would be considered an optimum concentration [13]. Such high sulfur content can cause several undesirable effects as the competition of sulfate-reduction with methanogenesis, reducing the conversion of organic acids into biogas, besides also negatively influencing the bioavailability of trace elements inside the bioreactors [23,24,27].

For the other sugarcane waste, sulfur and phosphorus contents were rather low. Scherer *et al.* [17] observations demonstrated that the addition of phosphate and sulphate increased considerably the degradation rate and biogas production from fodder beet silage as a mono-substrate, supporting the role of P and S as limiting macro nutrients for certain AD systems.

The development of a co-digestion strategy to balance the macronutrients of the sugarcane waste apparently could make sense from the economic point of view, once it could partially replace the addition of high cost chemicals as, for example, urea that usually would be used to balance the C:N ratio during AD of bagasse and straw. However, the co-digestion of vinasse with other lignocellulosic waste can also provide undesirable effects as increasing the production of H_2_S that could lead to the necessity of costly biogas desulfurization.

Moreover, another aspect that should be taken into account is the degradation rates of the substrates (see Subsection 2.2.2 and Subsection 2.2.3). The eventually co-digestion of waste with different degradation profiles could lead to a lower biomass conversion if the HRT is too short compared to the other co-substrate, or lead to an unnecessary increase in the reactor size if the HRT is based on the substrate with the slowest degradation rate.

#### 2.1.3. Trace Elements

A lack of some important trace elements can be seen from the composition of autonomous- and annexed-derived vinasse (Table 2); especially nickel is critical. Meanwhile, the concentrations of other trace elements, as tungsten, manganese, selenium, zinc, cobalt, molybdenum, and copper, are close to the lowest limit recommended by Kayhanian *et al.* [22] and Oechsner *et al.* [28].

**Table 2:** Trace elements content of the sugarcane waste.

Parameters	Units	Vinasse				Filter Cake (*n* = 9)		Bagasse (*n* = 9)		Straw (*n* = 12)		Recommendation	
		Autonomous (*n* = 6)		Annexed (*n* = 15)									
		AV	SD	AV	SD	AV	SD	AV	SD	AV	SD	[22]	[28]
Fe	mg·kg_TS_^−1^	200	±177	488	±142	27,267	±24,625	2012	±1530	14,949	±23,435	100–5000	750–5000
Ni	mg·kg_TS_^−1^	0.49	±0.03	2.30	±0.92	14.3	±5.81	4.04	±3.17	7.17	±5.22	5–20	4–30
Co	mg·kg_TS_^−1^	0.55	±0.02	0.62	±0.46	3.36	±1.34	0.52	±0.25	2.87	±3.73	<1–5	0.4–10
Mo	mg·kg_TS_^−1^	0.48	±0.01	0.84	±0.20	1.03	±0.74	0.58	±0.37	0.71	±0.25	<1–5	0.05–16
W	mg·kg_TS_^−1^	Nd	-	0.08	±0.04	0.29	±0.50	0.19	±0.05	0.24	±0.05	<1	0.1–30
Mn	mg·kg_TS_^−1^	59.6	±5.94	194	±49.2	566	±188	43.4	±11.1	177	±85.1	-	100–1500
Cu	mg·kg_TS_^−1^	3.62	±0.14	7.96	±4.03	43.8	±4.04	4.82	±1.93	10.7	±12.7	-	10–80
Se	mg·kg_TS_^−1^	Nd	-	0.08	±0.06	0.01	±0.02	0.83	±0.12	0.19	±0.24	-	0.05–4
Zn	mg·kg_TS_^−1^	36.8	±10.5	32.6	±4.45	132	±7.21	17.2	±10.1	10.1	±14.9	-	30–400

From the filter cake composition, less trace elements were observed below the recommendation values (*i.e.*, tungsten and selenium) in comparison to vinasse, as well as elements close to the lowest limit (*i.e.*, molybdenum). This occurs, probably, because at the industrial phase where filter cake is generated, metal based coagulants and flocculants are added during the physical-chemical process that removes impurities from sugarcane juice.

Trace element deficiencies were also found for bagasse and straw, as manganese, copper, zinc, tungsten, and selenium, as well as nickel, were found in a concentration close to the lowest recommended limit.

#### 2.1.4. Nutritional Values

Carbohydrates were the main constituents found in all analyzed samples, though differing considerably into its components (Table 3). Filter cake, bagasse, and straw presented higher average lignin content, 116, 124, and 162 g·kg_TS_^−1^, respectively, while samples of vinasse had much lower values, 34.3 up to 56.5 g·kg_TS_^−1^. According to previous studies [29,30,31,32] the presence of lignin in certain substrates can negatively influence the microbial access to cellulose and hemi-cellulose during the AD process. Such type of substrate is frequently submitted to physical, chemical or biological pre-treatment procedures to accelerate and enhance degradation in large-scale biogas plants.

Non-fiber carbohydrates (NFC), which are easy accessible to microbial degradation, were found in different concentrations among two of the lignocellulosic waste types (*i.e.*, filter cake and bagasse). The high variation found in bagasse (181 ± 216 g·kg_TS_^−1^) is explained by different milling efficiencies during the extraction of the juice at the sugarcane plants, whereas the differences found among the three analyzed samples of filter cake (118 ± 136 g·kg_TS_^−1^) can be explained by the use of different technologies (*i.e.*, rotary vacuum-drum filter or filter press) to recover sucrose during the process of juice treatment.

**Table 3:** Nutritional values of the sugarcane waste.

Parameters		Units	Vinasse				Filter Cake (*n* = 9)		Bagasse (*n* = 6)		Straw (*n* = 9)	
			Autonomous (*n* = 6)		Annexed (*n* = 6)							
			AV	SD	AV	SD	AV	SD	AV	SD	AV	SD
Raw protein	-	g·kg_TS_^−1^	137	±5.17	159	±11.5	124	±12.8	17.9	±3.89	27.7	±8.36
Raw fat	-	g·kg_TS_^−1^	0.72	±0.85	0.12	±0.10	39.5	±5.11	7.20	±3.27	9.18	±1.55
Carbohydrate	NFC ^a^	g·kg_TS_^−1^	372	±44.7	263	±69.9	118	±136	181	±216	107	±41.2
	Cellulose	g·kg_TS_^−1^	34.9	±11.7	60.9	±13.8	126	±19.2	357	±96.1	311	±88.1
	Hemi-cellulose	g·kg_TS_^−1^	101	±27.5	243	±176	164	±16.3	233	±93.1	227	±14.5
Lignin	-	g·kg_TS_^−1^	56.5	±15.5	34.3	±3.03	116	±27.1	124	±38.2	162	±26.2
Raw ash	-	g·kg_TS_^−1^	296	±82.0	238	±101	309	±125	78.3	±10.1	154	±120
TKN ^b^	-	g·kg_TS_^−1^	22.2	±0.78	49.6	±32.1	20.0	±2.09	2.90	±0.65	4.48	±1.36

^a^ non-fiber carbohydrates; ^b^ Total Kjeldahl nitrogen.

### 2.2. Biochemical Methane Potential

#### 2.2.1. Methane Yields

**Figure 1:**
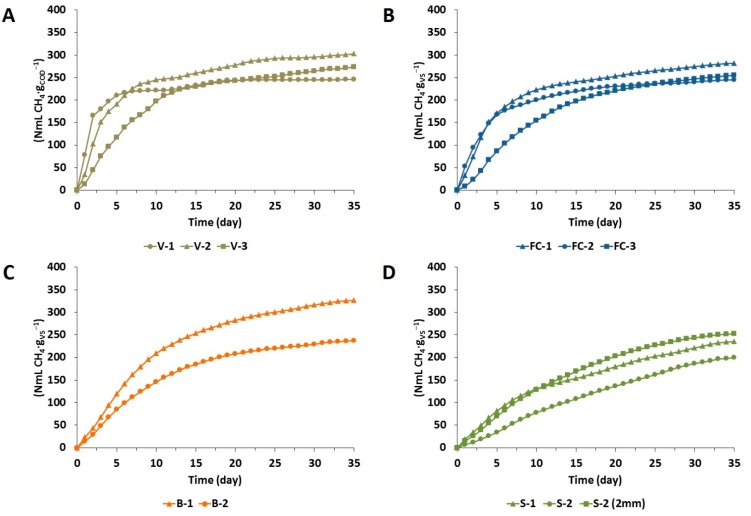
Cumulative methane yield of the sugarcane waste during 35 days of BMP assays. (**A**) vinasse from autonomous (V-1) and annexed plants (V-2 and V-3); (**B**) filter cake (FC-1, FC-2 and FC-3); (**C**) bagasse (B-1 and B-2); (**D**) straw (S-1 and S-2), and S-2 ground to 2 mm.

The results of BMP assays are presented in Figure 1 and Table 4. It can be observed that vinasse from the autonomous plant (V-1) achieved the lowest methane yield after 35 days (246 ± 15 NmL·g_COD_^−1^), in comparison to the two samples from the annexed plants (273 ± 02 and 302 ± 06 NmL·g_COD_^−1^). However, when these results are given in methane production per fresh matter, the differences among samples of autonomous and annexed plants are even higher. Sample V-1 achieved the methane yield of 5 Nm^3^·ton_FM_^−1^, while samples V-2 and V-3 achieved higher values, 7 and 11 Nm^3^·ton_FM_^−1^, respectively. The reason for such differences is due to the lower COD content found in the sample from the autonomous plant (23.1 g·L^−1^) in comparison to the samples from the annexed plants (24.9–39.5 g·L^−1^).

The methane yield achieved by the three filter cake samples did not present any major difference in terms of final methane yield, varying between 245–281 NmL·g_VS_^−1^. When the results are converted to methane production per fresh matter (50 up to 58 Nm^3^·ton_FM_^−1^), it becomes clear that filter cake has a higher energy content than vinasse from the autonomous plant (around 10 times higher), or even from the annexed plant (around 7 times higher).

However, in comparison to vinasse it seems that the higher lignin content of filter cake could have hampered the utilization of the cellulose and hemi-cellulose during the biogas conversion. This fact is even clearer in sample (FC-3), where the lower NFC content also influenced the lag phase during its degradation.

The methane yields obtained from two different samples of bagasse varied considerably between 236–326 NmL·g_VS_^−1^ (119 to 181 Nm^3^·ton_FM_^−1^), possibly due to different NFC concentrations found in these samples (181 ± 216 g·kg^−1^ TS). In a different way, samples of straw did not vary considerably into its nutritional content, however presented slightly different methane yields from 199 to 234 NmL·g_VS_^−1^ (101 to 126 Nm^3^·ton_FM_^−1^). Nevertheless, submitting the straw to a physical pre-treatment by grinding to 2 mm, resulted in 26% higher methane yield.

Therefore, the theory that smaller particle sizes increases the specific surface area of the biomass, providing greater possibility for enzymatic attack [33] is reinforced. Thus, if the straw would have undergone a more intensive physical pre-treatment, it could have produced higher biogas yields.

**Table 4:** Biochemical methane potential of the sugarcane waste after 35 days of assay.

Substrates	Samples	Methane Yield * [NmL·g_VS_^−1^or NmL·g_COD_^−1^]	Methane Yield [Nm^3^·ton_FM_^−1^]	*K* [day^−1^]
Straw	S-1	234 ± 03	101 ± 01	0.091
	S-2	199 ± 23	126 ± 15	0.075
	S-2 (2mm)	252 ± 02	160 ± 01	0.102
Bagasse	B-1	326 ± 04	181 ± 02	0.119
	B-2	236 ± 05	119 ± 02	0.124
Filter cake	FC-1	281 ± 04	58 ± 01	0.159
	FC-2	245 ± 01	50 ± 01	0.178
	FC-3	254 ± 08	54 ± 02	0.091
Vinasse	V-1	246 ± 15	05 ± 01	0.413
	V-2	302 ± 06	08 ± 01	0.209
	V-3	273 ± 02	11 ± 01	0.107

* Methane yield of vinasse is given in NmL·g_COD_^−1^.

#### 2.2.2. Reaction Rates

The decay constant (*k*-value) calculated with Equation (1) (see Subsection 3.3) is presented in Table 4. Samples of vinasse achieved the highest *k*-value (0.107–0.413), mainly by the lower lignin and higher NFC content found in this material. Interestingly, the sample from the autonomous plant (V-1) presented a higher *k*-value (0.413) than samples from the annexed plants. Vinasse from the annexed plant that produces ethanol from a mix of sugarcane juice and molasses (V-2) presented an intermediate *k*-value (0.209), while vinasse from the annexed plant that produces ethanol exclusively from molasses presented a lower *k*-value (0.107). According to Wilkie *et al.* [34] the concentration of sugars in molasses, through crystalinization and evaporation of cane juice, increases the content of more recalcitrant organics which remain in the vinasse after fermentation. This fact supports the presented results, since vinasse from molasses-based ethanol resulted in longer lag phases in comparison to vinasse from sugarcane juice-based ethanol.

Samples of filter cake achieved intermediate *k*-values (0.091–0.159), however, as well as for vinasse, one of the samples achieved a completely different performance. In that case, sample FC-3 was from a sugarcane plant that applies a different strategy during the juice treatment, where small pieces of bagasse are intentionally added to the filter cake to increase its permeability, enhancing the recovery of residual sucrose at the rotary vacuum-drum filter. Therefore, the longer lag phase can be explained by the lower NFC content found in this sample.

Although the samples of bagasse presented considerably different methane yields, both displayed similar *k*-values (0.119–0.124). In the meantime, the two different straw samples presented the slowest reaction rates (0.075–0.091) of all analyzed materials, even always presenting similar values of TS, VS, and nutritional values in comparison to bagasse. Fact that explained by the longer length of its fibers (±6 cm) in comparison to bagasse (±3 cm), once when the sample of straw (S-2) was submitted to a mechanical pre-treatment, the *k*-value increased from 0.075 to 0.102.

#### 2.2.3. Substrate Conversion in a CSTR System

The substrate conversion in a hypothetical CSTR system was simulated with Equation (2) and data presented in Table 5. Depending on the feedstock used for ethanol production, the HRT needed to achieve 80% of the vinasse degradation would vary considerably (10–40 days). In this case, the vinasse derived from an autonomous plant would demand a shorter HRT. On the other hand, the vinasse derived from an annexed plant that was using the entire sugarcane juice for sugar production at the time of sampling—that is, only molasses mixed with water was used for ethanol production—would demand a longer HRT. However, for the Brazilian conditions, where, in most cases, the ethanol is produced by a mix of molasses and juice, the most likely scenario would be the values found for vinasse (V-2).

In this way, due to economic reasons, one of the main concerns during the choice of an AD system is to reach the expected methane yield with maximum organic loading rate (OLR) and, consequently, minimum hydraulic retention time (HRT) that can be applied under stable process conditions.

Considering the low TS content of vinasse and the absence of clogging materials, it seems to be clear that the utilization of a biomass immobilization system would be the most suitable for this substrate, once it would permit a lower HRT in combination with higher solid retention times (SRT), considerably reducing the size of the reactors in comparison to the conventional continuous stirred-tank reactor (CSTR) system.

Therefore, the AD using vinasse as a substrate for biogas production should focus on these types of reactors, such as fixed bed reactors, fluidized bed reactors or granular sludge systems, especially the upflow anaerobic sludge blanket (UASB) reactor, as this system is already being successfully used in Brazil for the treatment of different types of wastewater. Thus, the process of technology scaling up could be facilitated, once the basic reactor concept and operation is already widespread in the region [35].

The results from simulating the filter cake samples also presented a wide range of HRT (25–45 days). This is justified since one of the samples (FC-3) has presented a longer lag phase and, consequently, lower *k*-value. The two bagasse samples presented the same conversion profile in general, demanding longer HRT in comparison to vinasse and filter cake, in a similar way to straw, that would need between 45–55 days to achieve 80% of its degradation. Nevertheless, the effect of a physical pre-treatment could increase the methane yield, or alternatively reduce the HRT from 55 to 40 days as demonstrated in the straw S-2 (2 mm).

**Table 5:** Simulation of the hypothetical substrates conversion using CSTR system.

HRT (Days)	Vinasse			Filter Cake			Bagasse		Straw		
	V-1	V-2	V-3	FC-1	FC-2	FC-3	B-1	B-2	S-1	S-2	S-2 (2 mm)
5	0.67	0.51	0.35	0.45	0.47	0.31	0.37	0.38	0.31	0.27	0.34
10	**0.80**	0.68	0.52	0.62	0.64	0.48	0.54	0.55	0.48	0.43	0.50
15	0.86	0.76	0.62	0.71	0.73	0.58	0.64	0.65	0.58	0.53	0.60
20	0.89	**0.81**	0.68	0.77	0.78	0.65	0.70	0.71	0.64	0.60	0.67
25	0.91	0.84	0.73	**0.80**	**0.82**	0.70	0.75	0.76	0.69	0.65	0.72
30	0.93	0.86	0.76	0.83	0.84	0.73	0.78	0.79	0.73	0.69	0.75
35	0.94	0.88	0.79	0.85	0.86	0.76	**0.81**	**0.81**	0.78	0.72	0.78
40	0.94	0.89	**0.81**	0.87	0.88	0.79	0.83	0.83	0.78	0.75	**0.80**
45	0.95	0.90	0.83	0.88	0.89	**0.80**	0.84	0.85	**0.80**	0.77	0.82
50	0.95	0.91	0.84	0.89	0.90	0.82	0.86	0.86	0.82	0.79	0.84
55	0.96	0.92	0.86	0.90	0.91	0.83	0.87	0.87	0.83	**0.80**	0.85
60	0.96	0.93	0.87	0.91	0.91	0.85	0.88	0.88	0.84	0.82	0.86
65	0.96	0.93	0.87	0.91	0.92	0.86	0.89	0.89	0.85	0.83	0.87
70	0.97	0.94	0.88	0.92	0.93	0.86	0.89	0.90	0.86	0.84	0.88

HRT = hydraulic retention time; The results highlighted in bold correspond to approximately 80% of the substrate conversion at different HRT.

### 2.3. Process Design

#### 2.3.1. Energy Potential

**Figure 2:**
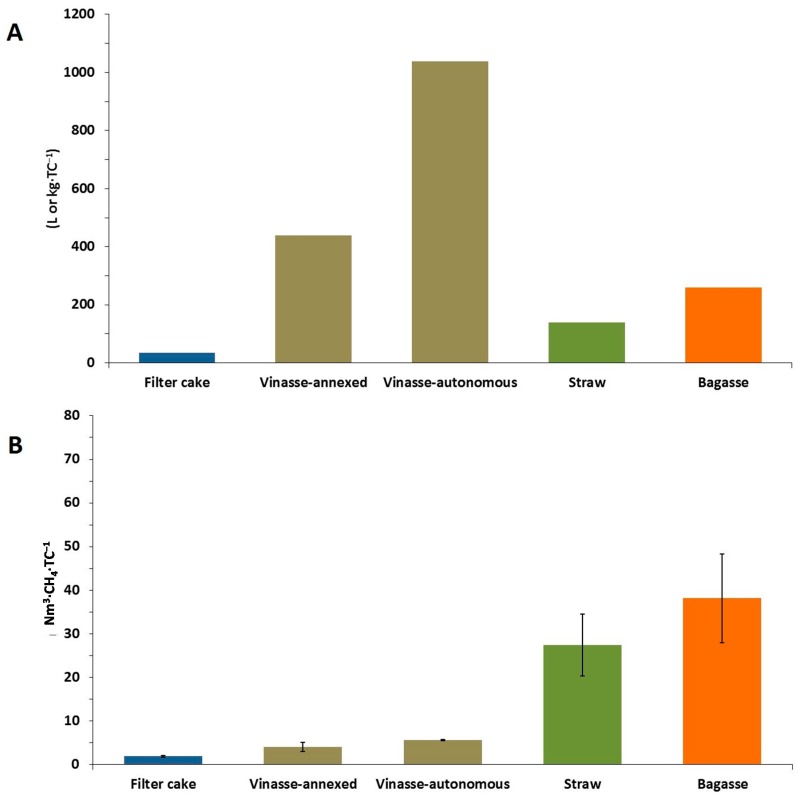
Mass flows VS energy potential of the sugarcane waste. (**A**) The values liters or kilogram per ton of cane (L or kg·TC^−1^) refer to fresh matter according to data collected in two different sugarcane plants, except straw (dry matter) according to previous study [4]; (**B**) The values normal cubic meter of methane per ton of cane (Nm^3^·CH_4_·TC^−1^) were calculated based on the BMP results and data presented in Figure 2A.

BMP results combined with specific waste generation from two different sugarcane plants (Figure 2A) were used to assess the energy potential in a hypothetical plant with a capacity to process 4 million tons of cane (TC) per year (Figure 2B).

Although vinasse from the autonomous plant has a lower methane yield per fresh matter, an autonomous plant could produce 40% more energy from vinasse in comparison to an annexed plant. This is possible due to differences in ethanol production between these two types of plants, from 30 to 71 L·TC^−1^, directly influencing the specific vinasse generation of them (Figure 2A). Even filter cake being produced in clearly lower amounts in comparison to vinasse derived from autonomous and annexed plants, the energy potential of filter cake is close to the vinasse potential. 

Although bagasse presented the highest methane potential, its entire utilization as a substrate for biogas production may suffer restrictions due to the fact that it is already being used for co-generation purposes by the thermochemical conversion system. Straw would not suffer the same restriction, since nowadays, in most cases, this material is left to decay on the fields due to the low incentives to produce bioelectricity from it.

Nevertheless, the dissemination of second-generation biofuels in the future can lead to an extreme competition for biomass among different available energy conversion pathways, whereas it is expected that the system providing not only higher energy production, but also higher environmental benefits, will have better possibilities to play an important role in the market.

#### 2.3.2. Energy Complementarities

In order to understand how the AD process could be designed to enhance the utilization capacity of a biogas plant integrated to the sugar and/or ethanol production processes (Table 6), it is reasonable to develop a storage system that would permit the use of filter cake, or even parts of bagasse and straw, during the sugarcane offseason, in the same way as is already practiced for the maize ensiling in Germany, for example.

**Figure 3:**
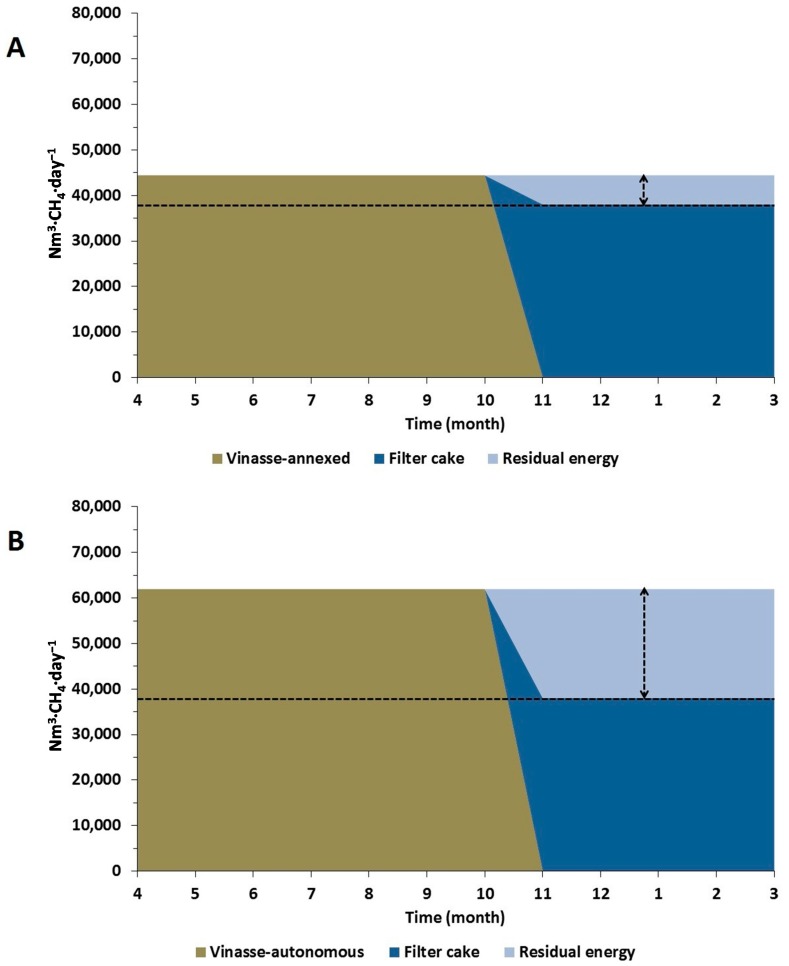
Energy potential of the vinasse and filter cake calculated for a hypothetical sugarcane plant. (**A**) Energy potential according to an autonomous sugarcane plant; (**B**) Energy potential according to an annexed sugarcane plant. Residual energy (highlighted by the dotted lines) is the difference in daily methane production between the potential energy of vinasse and filter cake.

The Figure 3 presents the daily methane generation that could be produced using vinasse during the 236 days of sugarcane season (±8 months, from April to November) and filter cake during the 129 days of offseason period (±4 months, from December to March) in the main sugarcane producing region in Brazil (*i.e.*, Center-South Region).

By applying this concept to an annexed plant, the daily methane production that filter cake during the offseason period could provide is 14.4% lower than the daily methane production of vinasse during the sugarcane season. The remaining methane production needed to compensate for such difference during the sugarcane offseason (6398 Nm^3^·CH_4_·day^−1^) could be provided by converting in methane 2.1% ± 0.5% of the total straw generated or, alternatively, 1.8% ± 0.4% of bagasse. In the meantime, when an autonomous plant is considered (Figure 3B), the remaining residual energy (23,925 Nm^3^·CH_4_·day^−1^) would be equivalent to 7.9% ± 1.9% of the energy potential straw or 6.8% ± 1.7% of bagasse.

For those plants where a fraction of straw or bagasse would not be available for biogas production, two different possibilities could be explored to keep methane production constant during the entire year.

Considering that, in Brazil, there is no environmental obligation that mandates the treatment of vinasse before fertirrigation on the fields, the biogas plant does not necessarily need to operate as a treatment facility and the amount of vinasse used for methane production could be reduced by 14.4% in the case of an annexed plant, and by 38.6% in the case of an autonomous plant. However, obviously in this case the final energy produced by the biogas plant would be reduced.

Another option could be the utilization of a pre-treatment process on filter cake to increase its specific methane production, since such an alternative has already been tested before. Gonzalez *et al.* [36,37] evaluated two different pre-treatment methods to increase the methane yields of filter cake (named as press mud) under mesophilic conditions (±37 °C). The liquid hot water pre-treatment method was able to increase the methane yield by 63% after 20 min of exposure at 150 °C, while the thermo-alkaline pre-treatment method was able to increase the methane yield by 72% by adding 10 g Ca(OH)_2_ per 100 g·TS^−1^ for 1 h. However, questions regarding the application of such methods at the large-scale still remain unclear, especially whether these types of pre-treatments would be able to provide net profit gain to the biogas system.

**Table 6:** Summary of the kinetic challenges for designing an anaerobic digestion process applied to the sugarcane industry.

Aspects	Vinasse	Filter cake	Bagasse	Straw
Reactor type	Biomass immobilization system (e.g., UASB)	CSTR or combination with biomass immobilization system	CSTR with high HRT (>35 days)	CSTR with high HRT (>40 days)
Pre-treatment	Not necessary	Recommended	Highly recommended	Highly recommended
Macronutrients	Phosphorous addition to balance C:P ratio at autonomous plant	Sulfur addition to balance C:S ratio	Nitrogen, sulfur and phosphorus addition to balance C:N:P:S ratio	Nitrogen, sulfur and phosphorus addition to balance C:N:P:S ratio
Trace elements	Lack of Fe, Ni, Co, Mo, W, Mn, Cu, Se, and Zn	Lack of Mo, W, and Se	Lack of Fe, Ni, Co, Mo, W, Mn, Cu, Se, and Zn	Lack of Fe, Ni, Co, Mo, W, Mn, Cu, Se, and Zn
Major challenge	High sulfur content, especially at annexed plants	Storage in case of use during sugarcane offseason	Low biomass availability; High lignin content;	Substrate logistic; High lignin content

## 3. Experimental Section

### 3.1. Substrates

Sugarcane waste derived from one autonomous plant, where ethanol is produced exclusively from the sugarcane juice, and sugarcane waste derived from two annexed plants, where ethanol is flexibly produced from molasses (*i.e.*, by-product from sugar production), sugarcane juice, or in most cases by a mix of both, were utilized in order to provide an extensive evaluation of the different existing sugarcane industrial processes found in Brazil. Therefore, samples of vinasse, filter cake, bagasse, and straw were collected from those sugarcane plants in the States of Goiás and São Paulo, Brazil during different seasons (2012–2013 and 2013–2014), transported to Germany in cooled boxes and kept under low temperature (*i.e.*, 4 °C) until its use.

### 3.2. Analytical Methods

For all samples, TS and VS were analyzed according to VDI 4630 [38]. For vinasse samples, chemical oxygen demand (COD) was also analyzed through LCK 014 COD kit (Hach-Lange, Düsseldorf, Germany) according to the manufacturer’s protocol.

Nutritional content of the substrates was determined according to Weender, followed by Van Soest methods. By the Weender method raw protein, raw fat, NFC, and raw fiber are determined. Van Soest method allows the determination of the remaining carbohydrates and lignin fractions from the neutral detergent fiber (NDF), which represents hemicellulose, cellulose, lignin and ash, acid detergent fiber (ADF) represented by cellulose, lignin and ash, and the lignin content depicted by the acid detergent lignin (ADL). Detailed description of the methods were previously published by Liebetrau [39].

To determine the major and trace elements contained in each sugarcane waste, dried samples were pretreated with a mixture of HNO_3_/H_2_O_2_/HF, followed by neutralization with H_3_BO_3_, and the resulting clear solution was analyzed by inductively-coupled plasma atomic spectrometry (ICP-OES, ThermoFischer iCAP6200) according to standard procedures [40,41,42].

### 3.3. Biochemical Methane Potential

The biogas yield of each sugarcane waste was obtained through BMP assays according to VDI 4630 [38] using eudiometer systems under mesophilic temperature (38 °C) for 35 days, and corrected to normal conditions, considered 273.15 K and 101.325 kPa. Methane concentration in biogas was measured by using a GA2000 Landfill Gas Analyzer (Geotechnical Instruments Ltda., Warwickshire, UK). The inoculum used for the BMP assays was originally from a large scale biogas plant, which uses maize silage and cattle manure as substrates. The decay constant (*k*-value) was calculated based on the results from BMP assays by using a first-order kinetic model (Equation (1)), which equates biogas production with organic mass reduction [43]:

(1) $$St=So\times \;e^{-kt}$$

where, *So*: initial mass of the substrate, substrate input (gVS); *St*: mass of the substrate at time *t*, after degradation (gVS); *k*: conversion rate (day^−1^); *t*: time (day).

In order to eliminate the lag phase and use only the log phase of the test, which better represent the first order kinetics, data between days 0–5, 0–12, 3–25, and 3–25 were considered for vinasse, filter cake, bagasse, and straw, respectively. The high values of coefficient of determination (*R*^2^), from 0.93 to 0.99, demonstrate the congruence of the model and experimental data.

The simulation of the substrates conversion using a CSTR system was performed by transferring the *k*-values previously calculated into the CSTR kinetics presented in Equation (2):

(2) $$St=\frac{So}{1\;+\;k\;\times \text{ θ }}$$

where, *So*: initial mass of the substrate, substrate input (gVS); *St*: mass of the substrate at time *t*, after degradation (gVS); θ: hydraulic retention time (day); *k*: conversion rate (day^−1^); *t*: time (day).

### 3.4. Energy Assessment

The energy potential of each sugarcane waste type was calculated based on the obtained BMP results, together with the specific waste production of annexed and an autonomous sugarcane plants. The energy complementarity between vinasse and the other types of waste was performed considering a hypothetical sugarcane plant with capacity to process 4 × 10^6^ tons of cane per year (TC·year^−1^), considering the same average days of operating season (236 days) as the two sugarcane plants previously analyzed.

## 4. Conclusions

The organic waste generated during the sugar and/or ethanol productions have different characteristics that should be taken into account during the design of the anaerobic digestion process. The challenges of straw and bagasse utilization as substrates for biogas production are clear as several potential nutritional deficiencies were identified for these substrates. Urea supplementation could not only balance the C:N ratio, but also increase the buffer capacity of the system and enhance the quality of the digestate for further use as fertilizer on the sugarcane fields. Although the higher methane yields of straw and bagasse (based on fresh matter), respectively, were 129 and 150 Nm^3^·ton_FM_^−1^, the high lignin content of these substrates suggests that a pre-treatment process can enhance their degradability. The lower methane yield of vinasse (5–11 Nm^3^·ton_FM_^−1^), together with its huge specific generation (438–1038 L·TC^−1^) suggests that future developments should be focused on a biomass immobilization reactor to allow higher OLR and lower HRT. Filter cake can play an important role to reduce the negative effects of sugarcane seasonality in the biogas system, if utilized as an alternative substrate to vinasse during the offseason. Such a concept still needs to be technically proven, especially regarding the feasibility of filter cake storage and the net energy gain that a pre-treatment procedure could provide to the system.
